# Solo Sonographically Guided PCNL under Spinal Anesthesia: Defining Predictors of Success

**DOI:** 10.1155/2016/5938514

**Published:** 2016-05-03

**Authors:** Akbar Nouralizadeh, Hamid Pakmanesh, Abbas Basiri, Mohammad Aayanifard, Mohammad Hossein Soltani, Ali Tabibi, Farzaneh Sharifiaghdas, Seyed Amir Mohsen Ziaee, Naser Shakhssalim, Reza Valipour, Behzad Narouie, Mohammad Hadi Radfar

**Affiliations:** ^1^Department of Urology, Shahid Labbafinejad Medical Center, Shahid Beheshti University of Medical Sciences (SBMU), Tehran, Iran; ^2^Department of Urology, Shahid Bahonar Hospital, Kerman University of Medical Sciences (KMU), Kerman 7613747181, Iran

## Abstract

*Aim*. Sonography has been brought in percutaneous nephrolithotripsy (PCNL) as an adjunct to or substitute for X-ray to restrict radiation exposure. This study was designed to investigate the possible predictors for the success of the solo sonographically guided PCNL.* Methods*. 148 consecutive cases were prospectively enrolled. All steps of PCNL were performed solely with sonography guidance under spinal anesthesia. Residual stones were evaluated the day after surgery using sonography and plain radiography.* Results*. The mean age was 46 ± 15 years; 40% of kidneys had hydronephrosis. The mean stone burden was 504 ± 350 mm^2^. The mean duration of surgery was 43 ± 21 minutes. The early stone-free rate was 92% in inferior or middle calyceal stones, 89.5% in single pelvic stones, 81.5% in partial staghorn stones, and 61.9% in staghorn stones. The mean residual stone size was 13 ± 8 mm. Logistic regression showed that a lower age and a larger stone burden significantly predicted positive residual stones. Fifteen percent of patients presented with grade I or II and six percent showed grade III complication based on Clavien classification. There was no cases of organ injury or death.* Conclusion*. Solo ultrasonographically guided PCNL under spinal anesthesia is feasible with an acceptable stone-free rate and complication rate.

## 1. Introduction

Various methods have been used to manage renal stones. Percutaneous nephrolithotripsy (PCNL) has been established as the gold standard for the treatment of large kidney stones [1]. PCNL is a minimally invasive procedure in which the access to the pelvicalyceal system (PCS) is achieved by the puncture and dilatation of the tract under fluoroscopic guidance. This step is an important one that can significantly affect the outcome of the procedure [2, 3].

Fluoroscopy is now routinely used as a guide for obtaining access to the calyceal system. However, radiation exposure is not harmless for patients, staff, and surgeons [4]. Radiation exposure during treatment of pediatric stone disease is not trivial and is significantly greater when PCNL is performed. Given the recommended maximum effective dose of 50 mSv in any one year, urologists should closely monitor the amount of fluoroscopy used and consider the potential for radiation exposure when choosing the operative approach [5]. To avoid this disadvantage efforts have been made to add ultrasound in the PCNL as an adjunct to [1] or a substitute [6–9] for radiation to reduce X-ray usage [5–10]. Ultrasonography removes the risk of radiation while it provides visual information about other body organs such as liver and spleen.

We designed this study to evaluate prospectively the success and complication rates of our method in completely ultrasonographically guided PCNL (UPCN). We attempted to determine which patient or stone characteristics are more appropriate for this technique.

## 2. Method

Patients with kidney stones larger than 2 cm or stones resistant to shock wave lithotripsy (SWL) were enrolled. All cases including single pelvis or calyceal, partial staghorn, and complete staghorn were included excepting patients with scattered stones in different calyxes, who were excluded. Patients with uncorrected coagulopathy were excluded. This study entailed all patients undergoing PCNL between January 2012 and June 2013 in Labbafinejad Medical Center, Tehran, Iran. Possible complications of the procedure were described and informed consent was obtained from all participants.

Two fellows, who were experts in fluoroscopy guided PCNL and had completed at least 30 cases of UPCN, performed all operations under the supervision of different attending staff members. For all patients spinal anesthesia was performed using bupivacaine while the patients were in lateral decubitus position and the side of surgery was positioned lower. After five minutes position was changed to supine and when level of anesthesia was established, the patients were positioned to lithotomy position. A Number 5 or 6 French ureteral catheter was inserted using cystoscopy. Then patients were positioned to the prone positon and the target calyx was accessed under the guide of ultrasound performed by the surgeon. A needle holder attached to the side of the probe was used. The target calyx was selected based on the stone location and burden. The probe was moved to detect the thinnest area of the cortex at the end of the desired calyx. The needle guide option of the ultrasonography was activated. In addition, the needle was observed during advancement to the desired calyx to make sure the needle tip does not bypass the calyx. When clear urine was aspirated, with or without saline pushed through the preinserted ureteral catheter, a 0.035′′ J-tipped guide wire was inserted into the calyx. A skin incision was performed and the Sheeba needle was removed. The depth of the needle penetration was measured—that is, the length from the skin to the tip of the needle—and all next steps were performed based on this measurement (tract length).

To compensate for kidney movement during tract dilatation, 15 millimeters was added to the measured tract length. However, in patients who had previously undergone open kidney surgery, we used the same length as the measured tract length. The fascia was dilated using a Number 9 French dilator. The antenna was inserted to the kidney on the guide wire and the tract was then dilated with a 28-F or 30-F Amplatz dilator in a single-stage dilatation technique. In this stage, the previously measured tract length as well as the tactile sensation of the surgeon was indicator of sufficient dilatation of the tract. Saline was injected through the ureteral catheter and the urine exit was checked from the dilator lumen in most cases. Then, an Amplatz sheath was inserted on the Amplatz dilator to the desired calyx based on tract length. Finally, the dilator and antenna were removed and nephroscopy was begun via the Amplatz sheath using a Number 24 F Richard Wolf*™* nephroscope. If the Amplatz sheath was placed out of the system, the guide wire was followed by a nephroscope, the parenchyma was dilated using stone forceps, and then the sheath was placed in the system over the nephroscope. Stones were fragmented with pneumatic Swiss LithoClast*™* and extracted using stone forceps. At the end of the procedure, ultrasonography was used to determine whether there were any residual stones. A nephrostomy tube was not placed routinely at the end of the operation, except in cases with considerable bleeding or significant injury to the pyelocaliceal system or if the ureteral catheter was not preinserted or was not entered into the pelvis. We barely placed a Double J (D-J) catheter in our patients.

The ureteral catheter was removed the day after surgery and the patient was discharged if no nephrostomy tube had been inserted. Other patients were discharged after nephrostomy removal on the second postoperative day if there were no complications. Plain radiography of the kidneys, ureters, and bladder (KUB), and kidney ultrasonography were used to detect the presence of any residual fragments on the first postoperative day and the stone-free rate (SFR) was calculated based on these data. To restrict radiation exposure, we did not perform CT scans to assess the residual stones. Complications were recorded based on Clavien classification [10].

### 2.1. Statistical Analysis

The stone burden was calculated based on the formula recommended by the European Association of Urology (stone area (mm^2^) = length*∗*wide*∗π∗*0.25 (*π* = 3.14159)) [11]. Logistic regression was performed to ascertain the effects of age, body mass index (BMI), gender, kidney side, hydronephrosis, stone burden and location, and a history of previous open or percutaneous surgery or SWL on the likelihood of positive residual stones. The chi-square test was performed to evaluate the differences in the SFR of the categorical variables. The SPSS software version 22 was used for data analysis. *p* values less than 0.05 were considered statistically significant.

## 3. Results

A total number of 148 patients (91 males and 57 females) were enrolled. Male patients were younger than females (*p* < 0.05), while female patients had a higher BMI (*p* < 0.001), Table 1. Most patients had partial staghorns (22%), and 20.6% had multiple stones in calyces and the pelvis; 15.6% had staghorn stones and a single pelvic stone was observed in 17% of patients. A single calyceal stone was present in 24.8% of the patients including 26 stones in the inferior calyx, 2 stones in the middle calyx, and 7 upper calyceal stones.

The mean operation time was 43 ± 21 minutes from which 12 ± 13 minutes were spent for access and dilatation and 31 ± 17 minutes for nephroscopy. On average, 3 ± 2 puncture attempts were made to obtain access to the target calyx. Most accesses were from the inferior calyx (55.6%). Operations were generally carried out through single access (94%). A nephrostomy tube was placed at the end of the operation in 31% of cases. The causes for placing a nephrostomy tube were injured systems (16 cases; 39%), hemorrhages (15 cases; 36%), a misplaced or no ureteral catheter in place in 15%, and infected systems (4 cases; 10%).

In eight cases (5%), the operation failed, including two operations with failed access to the calyceal system, three cases in which dilatation failed, and three with failure to find the stone during nephroscopy despite successful access. Female gender, a history of previous kidney surgery, right-sided stones, and an absence of hydronephrosis were more prevalent in the cases with failed operations. However, only the kidney side was statistically significantly associated with the risk of failure (75% occurred in the right kidney; *p* = 0.04), Table 3. We observed a higher rate of complications in patients with failed operation, including hemorrhage and prolonged leakage (*p* < 0.001).

### 3.1. Dilatation Success

After dilatation and the placement of the Amplatz sheath, in the beginning of the nephroscopy, in 55.9% of cases the Amplatz sheath was correctly placed in the pyelocaliceal system (successful dilatation). In others, however, the sheath was out of the system in 38.6% of cases or passed the system in 5.5% of cases (unsuccessful dilatation). In 97% of these patients, the surgeon was able to follow the guide wire and enter the calyx. Nonetheless, in 3% of these patients, the operation failed. This failure rate was similar to cases who had successful dilatation (5%). Finally, SFR was worse in patients with unsuccessful dilatation compared to patients with successful dilatation (66% compared with 83%; *p* = 0.02). However, the complication rate was similar.

The specificities of these patients and their stones were analyzed to determine in which situations there is greater susceptibility for unsuccessful dilatation. Younger patients were found to be prone to this complication (43 ± 13 years compared with 50 ± 12 years; *p* = 0.001). In addition, in patients with a previous PCNL surgery, unsuccessful dilatation occurred in only 17% of occasions, compared with 40% in patients who never had a history of PCNL (*p* = 0.07). There was no significant association between success of dilatation and history of open surgery or SWL, kidney side, BMI, presence of hydronephrosis, or stone burden and location. Moreover, neither the calyx accessed nor the Amplatz sheath size was related to this issue.

### 3.2. Stone-Free Rate

Excluding eight failed operations, postoperative imaging data were missing in nine cases. In the remaining 131 patients, in early postoperative imaging, a residual stone was detected in 36 patients (27.5%). The mean residual stone size was 13 ± 8 mm and the most common site for residual stones was in the inferior calyx (40%). The plan for residual stones was SWL in 22 patients, re-PCNL for four patients, and medical therapy for 10 patients who had small residues less than 5 mm. When these 10 patients with insignificant residues (less than 5 mm) were excluded, the overall early SFR was 80.2%. The best SFR results were obtained in single lower or middle calyceal stones (92%) and the weakest were for complete staghorn stones (61.9%), Table 4.

As described in Table 2, the presence of a significant residual stone in the early (24 hours) postoperative imaging was associated with a lower age and a larger stone burden (*p* = 0.002 for both). While the SFR was 97% in patients with stones less than 300 mm^2^; this rate decreased to 61.3% in stones larger than 701 mm^2^. The rate of residual stones was the same for both operating fellows. In addition, previous positive histories of an open or percutaneous stone surgery or SWL were not related to the SFR. Surprisingly, the absence of hydronephrosis was not associated with poor SFR results.

Logistic regression was performed to ascertain the effects of age, BMI, gender, kidney side, hydronephrosis, stone area, and a history of previous open or percutaneous surgery or SWL on the likelihood of positive residual stones early postoperatively. Finally, only age and stone burden significantly predicted residual stones. The model explained 29.0% (Nagelkerke *R* ^2^) of the variance in positive residual stones and correctly classified 78.4% of cases. A lower age and a larger stone burden were associated with an increased likelihood of exhibiting positive residues. When a cut-off limit of 55 years was considered, the SFR was 92.1% for older patients compared with 73.9% in younger ones (*p* = 0.01).

### 3.3. Complications

The mean hemoglobin drop was 2.2 mg/dL. A postoperative creatinine rise of more than 0.5 mg/dL occurred only in three cases; of them, two cases had serum creatinine levels of more than 2 mg/dL. In one of them, a D-J catheter was inserted on the third postoperative day and the other cases were managed conservatively.

Fifteen percent of patients presented with grade I or II complications, while grade III complications occurred in 6%, based on Clavien classification. There were no cases of organ injury or death, Table 5.

## 4. Discussion

In the present study, we reported acceptable success and complication rates for our method in solo ultrasonographically guided PCNL (UPCN) with no C-arm available in the operating room. The overall SFR was 80.2%. It should be emphasized that we have reported the early (24 hours) postoperative SFR, which is usually higher than the typical SFR (after 30 or 90 days with or without secondary procedures) that is regularly reported in the literature. Indeed, many of the residual stones are small and may pass spontaneously. Similar results have been reported in large series of UPCN [9].

Several previous studies have reported acceptable results for PCNL under spinal or epidural anesthesia [9, 12]. The main advantage of spinal anesthesia seems to be the cooperation of the patient for positioning, especially in obese patients. Further, for high-risk patients, spinal anesthesia is safer than general anesthesia.

Although UPCN has been performed in flank [7] and supine [13] positions, in this study we preferred the prone position because kidney movement is restricted in this position compared with the lateral or supine position. However, we found that, in patients with no history of kidney surgery, the kidney is more mobile and the depth of the kidney during dilatation is actually greater than the tract length measured using the length of Sheeba needle penetration. In our practice, we added 15 mm to the tract size to compensate for kidney movement during blunt tract dilatation. Tract dilatation in the UPCN is not as accurate as fluoroscopy guided PCNL and it is more dependent on the surgeon's tactile sensation and skill. In fact, in 37% of circumstances, after removing the Amplatz dilator and initiation of the nephroscopy, we noticed that the Amplatz sheath was positioned out of the desired calyx. In this situation, we followed the guide wire and successfully fulfilled the operation in 97% of cases. It seems that previously operated upon kidneys are relatively fixed during the dilatation process and thus have less movement than non-operated upon kidneys and this problem occurred in only 17% of cases compared with 37% in all. Younger patients were more prone to this complication. To reduce this problem, Yan et al. [9] proposed a two-step dilation method in which they first placed a 16 F sheath in the system and adjusted the position of the sheath under direct vision using a 9.8 F nephroscope. Then, they dilated the tract further to place a 24 F sheath and performed nephroscopy using a 20 F nephroscope. Comparing our results with their study, the mean operating time was 43 min on average, compared with 66 min in their study. The cause of this difference may be that we used a larger nephroscope (24 F standard Richard Wolf nephroscope) and a larger Amplatz sheath (30 F dilator and 34 F sheath), which prepares better irrigation, and a larger working channel. Overall, the early SFR in our study was 80.2% compared with 63% in their report. However, for surgeons who have less experience with nephroscopy, especially at the beginning of the learning curve, managing an Amplatz sheath, in case it is placed out of the system, may be an obstacle and this is a drawback of this technique that may limit its popularization. A randomized clinical trial showed that there is no significant difference between sonographically guided access compared with fluoroscopically guided access for PCNL performed by trainee urologists in terms of intra- and postoperative parameters [14]. In our experience, beginner fellowships do well with this technique. Although the dilatation process is highly related to the experience, obtaining access to the desired calyx with Sheeba needle is significantly simpler to learn compared with fluoroscopically guided access.

### 4.1. Comparing UPCN with Fluoroscopy Guided PCNL

The standard approach for PCNL is considered fluoroscopically guided PCNL yet. However, there are many advantages of UPCN including reducing radiation exposure to surgeon, patient, and operating staff, particularly children and pregnant women, preventing adjacent organ damage, better localization of the radiolucent renal stones, and salvage for cases of failed retrograde ureteral stenting or failed or lost first access [15]. With appropriate training, ultrasound-guided renal access for PCNL can lead to reduced radiation exposure, accurate renal access, and excellent stone-free success rates and clinical outcomes [16]. A meta-analysis on 3019 patients including 1574 cases with sonography access and 1445 with fluoroscopic access revealed many advantages in favor of the sonographically accessed arm. These advantages were shorter access time, reduced intraoperative blood loss, a lower rate of operative complications, a lower rate of blood transfusion, and a higher stone-free rate [17].

In case of nonopaque stones, residual stone fragments could not be evaluated intraoperatively using fluoroscopy. By contrast, ultrasound could detect both opaque and nonopaque stones. However, there are several limitations. First, in contrast with the fluoroscopy, when a residual stone is detected by sonography, its position in relation to the nephroscope could not be assessed directly. Therefore, the stone clearance is more dependent on the surgeon's knowledge in calyceal anatomy as well as his/her skill in nephroscopy. Second, it is difficult to assess for residual stones during operation because, after fluid leakage, the kidney is pushed forward and fluid collected in the retroperitoneum may produce a posterior enhancement artifact resembling stone fragments. Nevertheless, for nonopaque stones, ultrasound is superior to fluoroscopy in terms of residual stone detection.

It is not rare for the urologist to lose the first access to the calyx. In this situation, in our experience, it is somewhat more difficult to obtain second access since the hydronephrosis is no longer present. Furthermore, the surgeon may erroneously obtain an access to the liquid accumulated in the retroperitoneum instead of calyceal system. However, we believe that, compared with fluoroscopy, second access is more feasible with ultrasound, especially for nonopaque stones. In this situation, contrast material injected through ureteral catheter spreads around the kidney and obtaining a secondary access using fluoroscopy guide is usually impossible. In these occasions, UPCN can have a roll as salvage procedure.

In the case of a superior calyx stone, the shadow artifact of the rib makes it extremely difficult to obtain intercostal access with a suitable angle to the desired calyx using ultrasound. Indeed, difficulties in obtaining intercostal access may be one of the drawbacks of UPCN. Still, in all cases of superior calyceal stones, we obtained subcostal access and our success rate was similar with cases of inferior or middle calyceal stones.

The risk of radiation is especially bold for young children or pregnant women. Sharifiaghdas et al. reported their experience with totally ultrasonography-guided PCNL using adult size instruments in children. They reported proper results and acceptable complications compared with the standard technique of PCNL. Likewise, this alternative method has the advantage of preventing radiation hazard in this age group [18].

We excluded cases with multiple scattered radiopaque stones because it is difficult to explore the kidney to find small stones scattered through the kidney using ultrasonography alone and the risk of positive residual stone is high. Combined ultrasonography and fluoroscopy is superior in these cases. In other cases including single pelvis or calyceal stones or partial or even complete staghorn stones our results with UPCN are comparable with reports of fluoroscopically guided PCNL.

A randomized clinical trial should be planned to compare the success and complication rates of UPCN with standard fluoroscopy guided PCNL thoroughly. Basiri et al. performed a randomized trial comparing these two approaches. After completion of the ultrasonographically guided access, they checked the accuracy of their access using fluoroscopy [19]. In fact, they did not accurately evaluate solo ultrasonographically guided PCNL, as when fluoroscopy is available, the surgeon may change the position of the sheath after obtaining sonographically guided access. Therefore, that success rate should not be extrapolated to cases in which the C-arm is not present in the operating room. In addition, they did not pursue the main goal, which is “to eliminate the radiation exposure to the patient and operating staff.” The worse thing is that heavy lead aprons for the surgeon are yet necessary. Falahatkar et al. reported their results of a clinical trial with the same method and did not use confirmatory fluoroscopy in the ultrasonography-guided group [13]. The sample size of this study was limited, but they reported similar results in both groups.

## 5. Conclusion

Solo ultrasonographically guided PCNL under spinal anesthesia is feasible with an acceptable SFR and complication rate. Inferior results were obtained for younger patients and larger stones.

## Figures and Tables

**Table 1:** Demographic data and specificities of the stone in men and women.

	Gender			
	Male (*n* = 91)		Female (*n* = 57)	
Age (years)	45.3 ± 13.6		51.8 ± 12.3	
BMI (Kg/m^2^)	25.6 ± 3.8		29.0 ± 4.8	
History of open surgery				
Yes	12	13.30%	12	21.10%
History of PCNL				
Yes	9	10.00%	8	14.00%
History of SWL				
Yes	8	13.10%	5	12.20%
Side				
Right	32	37.20%	25	46.30%
Left	54	62.80%	29	53.70%
Hydronephrosis				
No	48	58.50%	34	63.00%
Yes	34	41.50%	20	37.00%
Opacity				
Nonopaque	13	15.10%	14	25.00%
Opaque	73	84.90%	42	75.00%
Stone area (mm^2^)	537.5 ± 408.3		455.4 ± 275.7	
Stone area subgroup				
A (≤30 mm^2^)	0	0.00%	0	0.00%
B (31–300 mm^2^)	22	24.40%	14	25.00%
C (301–700 mm^2^)	46	0.5	32	0.6
D (>701 mm^2^)	22	0.2	10	0.2

Data are presented as mean ± standard deviation or count and column percent.

**Table 2:** Data of patients regarding their residual stone status in early postoperative imaging, excluding eight failed operations.

	Significant residue				*p* value
	No (105)		Yes (26)		
Age (years)	50 ± 14		41 ± 12		**0.002** ^∗^
BMI (Kg/m^2^)	27 ± 4		27 ± 5		0.67
Gender					
Male	64	80.0%	16	20.0%	0.95
Female	41	80.4%	10	19.6%	
History of open surgery					
No	91	81.3%	21	18.8%	0.44
Yes	14	73.7%	5	26.3%	
History of PCNL					
No	93	80.2%	23	19.8%	0.98
Yes	12	80.0%	3	20.0%	
History of SWL					
No	62	78.5%	17	21.5%	0.33
Yes	10	90.9%	1	9.1%	
Side					
Right	39	81.3%	9	18.8%	0.75
Left	60	78.9%	16	21.1%	
Hydronephrosis					
No	59	84.3%	11	15.7%	0.25
Yes	38	76.0%	12	24.0%	
Stone area (mm^2^)	469 ± 372		696 ± 363		**0.007** ^∗^
Stone area subgroup					
A (≤30 mm^2^)	0	0.0%	0	0.0%	**0.002** ^∗^
B (31–300 mm^2^)	32	97.0%	1	3.0%	
C (301–700 mm^2^)	53	80.3%	13	19.7%	
D (>701 mm^2^)	19	61.3%	12	38.7%	
The calyx accessed to					
Inferior	60	83.3%	12	16.7%	0.72
Middle	31	77.5%	9	22.5%	
Superior	11	73.3%	4	26.7%	
Pelvis	1	100.0%	0	0.0%	
Situation of the Amplatz sheath in the beginning of nephroscopy					
Out of the system	33	68.8%	15	31.3%	**0.03** ^∗^
In the system	65	87.8%	9	12.2%	
Over (passed the system)	6	85.7%	1	14.3%	

Data are presented as mean ± standard deviation or count and row percent.

^*∗*^Significant.

**Table 3:** Data of patients with failed operations compared with successful operations.

	Successful (*n* = 140)		Failed (*n* = 8)		*p* value
	Count	Column *N* %	Count	Column *N* %	
Gender					
Male	88	62.9%	3	37.5%	0.15
Female	52	37.1%	5	62.5%	
Previous surgery in this kidney					
No	106	76.3%	4	50.0%	0.09
Yes	33	23,7%	4	50.0%	
Side					
Right	51	38.6%	6	75.0%	**0.04** ^∗^
Left	81	61.4%	2	25.0%	
Hydronephrosis					
No	76	59.4%	6	75.0%	0.38
Yes	52	40.6%	2	25.0%	
Clavien score for complications					
I	5	3.6%	0	0.0%	**<0.001** ^∗^
II	21	15.0%	1	12.5%	
III	7	5.0%	4	50.0%	
IV	0	0.0%	0	0.0%	
V	0	0.0%	0	0.0%	

Data are presented as count and column percent.

^*∗*^Significant.

**Table 4:** Early SFR in relation to the stone location.

	Count	Early SFR
Multiple stones	26	80.8%
Single pelvic stone	19	89.5%
Complete staghorn	21	61.9%
Partial staghorn	27	81.5%
Inferior or middle calyx	25	92.0%
Superior calyx	6	83.3%

**Table 5:** Rate of complications including 140 successful and 8 failed cases.

Clavien score for complications	Frequency	Percent
*I (deviation from normal course without the need for intervention)*	*5* ^*∗*^	*3.4%*
Fever	6^*∗*^	4.1%
Transient elevation of serum Cr	3	2.0%

*II (minor complication requiring intervention)*	*22* ^*∗*^	*14.9%*
Blood transfusion	17^*∗*^	11.5%
Infections requiring additional antibiotics	9	6.1%

*IIIa (complications requiring intervention without general anesthesia)*	*5*	*3.4%*
D-J catheter placement for leak of more than 24 hours	3	2.0%
D-J catheter placement for UPJ or pelvis injury	1	0.7%
Retention and colic due to blood clots	1	0.7%

*IIIb (complications requiring intervention with general anesthesia)*	*6*	*4.1%*
Ureter-bladder stone	3	2.0%
AV fistula	1	0.7%
Perioperative bleeding requiring quitting the Op.	2	1.4%

*IV (life-threatening complications requiring IC management)*	*0*	*0.0%*

*V (death)*	*0*	*0.0%*

*Total*	*38*	*25.0%*

Subcategories whose frequency was null are not presented in the table.

^*∗*^Patients were categorized in the highest grade of complication if more than two complications were present.

## Abbreviations

BMI: Body mass index
CT-scan: Computed tomography scan
D-J: Double J catheter
PCNL: Percutaneous nephrolithotripsy
SFR: Stone-free rate
SWL: Shock wave lithotripsy
UPCN: Ultrasonographically guided percutaneous nephrolithotripsy.
